# The Sonic Hedgehog signaling pathway regulates autophagy and migration in ovarian cancer

**DOI:** 10.1002/cam4.4018

**Published:** 2021-06-02

**Authors:** Yibin Pan, Jiena Zhou, Weidan Zhang, Lili Yan, Meifei Lu, Yongdong Dai, Hanjing Zhou, Songying Zhang, Jianhua Yang

**Affiliations:** ^1^ Assisted Reproduction Unit Department of Obstetrics and Gynecology Sir Run Run Shaw Hospital Zhejiang University School of Medicine Zhejiang University Hangzhou China; ^2^ Key Laboratory of Reproductive Dysfunction Management of Zhejiang Province Sir Run Run Shaw Hospital Zhejiang University School of Medicine Zhejiang University Hangzhou China; ^3^ Department of Obstetrics and Gynecology Yaojiang Township Central Hospital Zhuji City Zhejiang Province China; ^4^ Department of Obstetrics and Gynecology Taizhou Hospital of Zhejiang Province Zhejiang University Taizhou City Zhejiang Province China; ^5^ Beilun district hospital of traditional Chinese medicine Ningbo city Zhejiang Province China; ^6^ Department of Pharmacy The Children's Hospital Zhejiang University School of Medicine National Clinical Research Center for Child Health Hangzhou Zhejiang Province China

**Keywords:** autophagy, chloroquine, cyclopamine, migration, sonic hedgehog pathway, xenografts

## Abstract

**Background:**

The Sonic Hedgehog (SHH) signaling pathway plays an important role in various types of human cancers including ovarian cancer; however, its function and underlying mechanism in ovarian cancer are still not entirely understood.

**Methods:**

We detected the expressions of SHH and SQSTM1 in borderline ovarian tumor tissues, epithelial ovarian cancer (EOC) tissues and benign ovarian tumor tissues. Cyclopamine (Cyp, a well‐known inhibitor of SHH signaling pathway) and chloroquine (CQ, the pharmaceutical inhibitor of autophagy) were used in vivo and in vitro (autophagic flux, CCK‐8 assay, wound healing assay, transwell assay, tumor xenograft model). The mechanism of action was explored through Quantitative RT‐PCR and Western Blot.

**Results:**

We found up‐regulation of SHH and accumulation of SQSTM1/P62 in epithelial ovarian cancer. Cyp induced autophagy through the PI3K/AKT signaling pathway. Moreover, low‐dose Cyp and chloroquine (CQ) significantly promoted the migratory ability of SKOV3 cells.

**Conclusions:**

Our findings suggest that inhibition of the SHH pathway and autophagy may be a potential and effective therapy for the treatment of ovarian cancer.

## Graphical abstract

In this manuscript, we show that SHH signaling was active in ovarian cancer tissues. Inhibition of SHH signaling by cyclopamine (Cyp) enhanced the autophagy and promoted autophagic flux in ovarian cancer cell lines through PI3K/AKT signaling pathway. Our findings provided the experimental basis for simultaneously inhibiting the SHH pathway and autophagy as a new effective therapeutic strategy for patients with ovarian cancer.

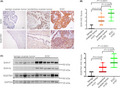

## 1 INTRODUCTION

Ovarian cancer (OC) is the most common gynecological cancer and is the leading cause of cancer‐associated mortality worldwide.^1^, ^2^ Although many new advancements have been made, the 5‐year survival rate for patients with ovarian cancer is a miserable 46%, due to the recurrence and severe metastasis.^3^, ^4^, ^5^ Therefore, novel therapeutic approaches to improve patient outcomes are urgently needed.

The Sonic Hedgehog (SHH) signaling pathway is vital for embryonic development and tissue homeostasis, and aberrant activation of this pathway is closely related to tumorigenesis and progression of many types of human cancer including ovarian cancer.^6^, ^7^, ^8^ The SHH signaling pathway consists of patched (Ptch/Ptch1), smoothened (SMO), and GLI (glioma‐associated oncogene transcription factors). Once the SHH ligand bind to Ptch1, the inhibition of Smo by Ptch is relieved, and then facilitates the post‐translational process of the transcription factor GLI, which are closely related to cancer cell survival, cell growth, and epithelial‐mesenchymal transition (EMT) through regulating expressions of downstream target genes.^9^, ^10^ Recent clinical studies demonstrated that targeting SHH signaling pathway is beneficial for various types of human cancers.^11^, ^12^ For example, vismodegib and sonidegib, two SHH pathway inhibitors, are being used in phase I and phase II clinical trials in basal cell carcinoma.^13^ In addition to these SMO inhibitors, GLI1 inhibitors including GANT58 and GANT61 are in pre‐clinical trials in breast cancer.^14^ For ovarian cancer, inhibiting the SHH signaling pathway by cyclopamine (Cyp), an inhibitor of the SHH pathway, resulted in the reduction of ovarian cancer cell viability, as well as arresting the tumor growth in vivo.^15^ This highlights that inhibition of the SHH pathway may serve as a target for the treatment of ovarian cancer.

Autophagy is a conservative metabolic process during which several cellular components including damaged organelle or misfolded proteins were degraded and recycled by lysosomes.^16^, ^17^, ^18^ Mounting evidence has demonstrated the predominant role of autophagy in the progression of human cancers.^19^, ^20^ For example, Akin et al. showed that autophagy antagonist, NSC185058 effectively suppressed autophagy in vivo and the growth of osteosarcoma tumors in pre‐clinical xenograft models.^21^ McAfee et al. found that effective low dosages of Lys05 (a novel autophagy inhibitor) inhibited tumor growth by inducing cell apoptosis and autophagy in mice.^22^ Autophagy is controlled by a highly regulated set of signaling events, occurs at a basal level in all cells, and is induced by diverse signals and cellular stresses. Recently, several studies have indicated that the SHH signaling pathway regulates autophagy in human cancers, and such interactions exhibit distinct pathological or pharmacological roles.^13^, ^23^ For example, SHH signaling represses autophagy to suppress tumor growth in stromal stellate cells by subduing autophagy‐induced alanine secretion.^24^ It has also been reported that inhibition of the SHH pathway by GANT61 inhibited tumor formation and decreased tumor volume through induction of autophagy by up‐regulation of Bnip3 in hepatocellular carcinoma cell (HCC).^25^ However, the interaction between the SHH pathway and autophagy in ovarian cancer remains unclear.

In this study, we discussed the activation of the SHH pathway and autophagy in ovarian cancer and further examined the influence of the SHH signaling pathway on autophagy. Moreover, the underlying molecular mechanism involved in the oncogenic functions of the SHH pathway was investigated. Our findings provide a theoretical basis for simultaneously inhibiting the SHH pathway and autophagy in the treatment of ovarian cancer.

## 2 MATERIALS AND METHODS

### 2.1 Patients and samples

Twenty one benign ovarian tumor tissues, 20 borderline ovarian tumor tissues, and 57 EOC tissues were obtained from patients who underwent ovarian resection at the Sir Run Run Shaw Hospital, School of Medicine, Zhejiang University between January 2015 and December 2018. Written informed consent was received by each patient and our research was approved by the Ethics Committee of the Sir Run Run Shaw Hospital, School of Medicine, Zhejiang University. The study protocol adhered strictly to the Code of Ethics of the World Medical Association (i.e., Declaration of Helsinki). All patients were over 18 years old and did not undergo radiotherapy, chemotherapy, or targeted therapy prior to surgery, with sufficient clinical data. All tissue specimens and slides were reevaluated by qualified gynecology pathologists. All samples were frozen at −80℃ for further analysis.

### 2.2 Immunohistochemistry staining

Tumor tissues were treated with a fixative solution for 24 h at 4℃ (4% formaldehyde and 1% glutaraldehyde in PBS, pH 7.4). They were then embedded in paraffin and then cut into 5 μm sections using a Leica SM2010R microtome (Leica). Subsequently, the paraffin sections were used for immunohistochemistry staining (IHC) as previously described.^26^ Briefly, these sections were deparaffinized and rehydrated, before they were boiled in Sodium Citrate Buffer (10 mM Sodium Citrate, 0.05% Tween 20, pH 6.0). Endogenous peroxide activity was then blocked by incubating slides with 0.3% hydrogen peroxide in methanol for 20 min. Then, the sections were blocked with 10% bovine serum for 30 min and incubated overnight at 4℃ with specific antibodies SHH (cat no. ab53281, Abcam, dilution 1:100) and SQSTM1 (cat no. ab56416, Abcam, dilution 1:100). Then the sections were incubated with EnVision™ Detection Kit, Peroxidase/DAB, Rabbit/Mouse (GK500710) and counterstained with hematoxylin (Fisher Scientific Company). Images were captured with the microscope (magnification ×200) and scores were given as previously described^27^ with some modification. The scores were calculated by multiplying intensity by positive staining rate and showed a range from 1 (negative) to 5 (strong).

### 2.3 Cell culture and reagents

Human ovarian cancer cell lines; SKOV3 (310551, BNCC), A2780 (310551, BNCC), and 3AO (338624, Fenghui Biotechnology Co, Ltd) were used in this study. They were cultured in RPMI 1640 (Gibco, Thermo Fisher Scientific), supplemented with 10% fetal bovine serum (FBS, Gibco) at 37℃ and 5% CO_2_. DNA (STR) profiling analysis was performed to authenticate these cell lines, and mycoplasma contamination was assessed. Cyclopamine (Cyp) (cat no. S1146) and AMPK inhibitor Compound C (Comp C) (cat no. S7306) were acquired from Selleck Chemicals. Chloroquine (CQ) (cat no. C6628) was obtained from Sigma‐Aldrich. Ad‐mCherry‐GFP‐LC3B was purchased from Han Bio‐Technology (HB‐AP210 0001).

### 2.4 Cell proliferation

The cytotoxicity effect of Cyp against SKOV3 cells was measured by using counting kit‐8 (CCK‐8, Dojindo). SKOV3 cells were seeded in 96‐well plates at 2500 cells/well overnight and then treated with different concentrations of Cyp for 24 h. After that, 10 μl CCK‐8 was added to each well and incubated for another 4 h. Then the absorbance of the samples at 450 nm was detected by the microplate reader (Bio‐Rad). All experiments were performed in triplicate.

### 2.5 Ad‐mCherry‐GFP‐LC3B transfection

When SKOV‐3 cells in 35‐mm plates grown to about 70% confluence, Ad‐mCherry‐GFP‐LC3B adenovirus was added into SKOV3 cells at a MOI of 80 for 24 h at 37℃. Following indicated treatment, autophagy was observed under Olympus BX53 fluorescence microscope. All experiments were performed in triplicate.

### 2.6 Wound healing assay

After SKOV3 cells reached 70% to 80% confluence, the cells were treated with CQ and/or Cyp for 24 h, and then the cell monolayer was scratched with a 10 μl pipette tip, and the scratches were photographed by a microscope (Carl Zeiss MicroImaging GmbH) (Magnification ×100) just after the denudation (time, 0 h). Cells were then incubated for another 12 and 36 h, and images were again recorded (time, 12 and 36 h). Image analysis was conducted with Image Analysis Software, V1.46 (National Institutes of Health). All experiments were performed in triplicate.

### 2.7 Transwell assay

Transwell chambers (8‐μm pore; BD Biosciences) coated with Matrigel (BD Biosciences) were used for invasion assay. Briefly, a total of 8 × 10^4^ SKOV3 cells were added in the top chamber, while the lower chamber was added with RPMI 1640 containing 20% FBS. After incubation for 24 h, the cells were stained with 0.1% crystal violet and photographed with an inverted microscope (Primovert, Carl Zeiss) at 200 ×  magnification. All experiments were performed in triplicate.

### 2.8 Western blot

Western blot was performed as previously described.^26^ Briefly, cells from 12‐well and 50–100 mg tissue were lysed with the RIPA (P0013B, Beyotime Biotechnology) and quantified with a BCA protein assay kit (Pierce; Thermo Fisher Scientific, Inc.). 40 μg protein samples were separated by 12% SDS‐PAGE and then transferred onto a polyvinylidene difluoride (IPVH00010, Millipore) membrane at 200 mA for 1 h on ice. Membranes were blocked in 2% non‐fat milk and incubated with primary antibodies against anti‐Sonic Hedgehog (1:1000, cat no. ab53281, Abcam), LC3B (1:2500, cat no. L7543, Sigma‐Aldrich), p‐AKT T308 (1:1000, cat no. 13038, Cell Signaling), p‐AKT S473 (1:1000, cat no. 4060, Cell Signaling), AKT (1:1000, cat no. 4691, Cell Signaling), SQSTM1/P62 (1:1000, cat no. ab56416, Abcam), AMPK (1:1000, cat no. ab133448, Abcam), p‐AMPK (1:1000, cat no. 2535, Cell Signaling) and GAPDH (1:1000, cat no. 600004‐1‐Ig, Proteintech) at 4℃ overnight, followed by anti‐rabbit and anti‐mouse second antibodies (cat no. ab216773 and ab216776, Abcam) at a 1:10,000 dilution for 1 h at room temperature. The signals were visualized with Odyssey Infrared Imaging System and quantified using Image J software V1.46 (National Institutes of Health). All experiments were performed in triplicate.

### 2.9 Quantitative *RT‐PCR*

Total RNA was isolated by using TRIzol reagent (15596026, Invitrogen) according to the manufacturer's instructions. Purified total 1 μg RNA was cleaned of genomic DNA and reverse‐transcribed into cDNA using random primers with a HiScript^®^ II Q RT SuperMix for qPCR (+g DNA wiper) kit (R223‐01, Vazyme) under reaction conditions of 50℃ for 15 min and 85℃ for 5 s. Then, *qRT‐PCR* was performed using a SYBR green PCR kit on a real‐time PCR detection system (LightCycler 480, Roche). The amplification program was 40 cycles of the following: 95℃ for 15 s, 58℃ for 30 s, and 72℃ for 30 s. Primers were used as follows: *PTCH1* Forward 5’‐ACCAAGTGATCGTGGAAGCC‐3’, Reverse 5’‐GTGGGTGATGCCTGGATTCG‐3’; *Gli1* Forward 5’‐GGGTGCCGGAAGTCATACTC‐3’, Reverse 5’‐GCTAGGATCTGTATAGCGTTTGG‐3’; *SQSTM1* Forward 5’‐AGGGAACACAGCAAGCT‐3’, Reverse 5’‐GCCAAAGTGTCCATGTTTCA‐3’; *GAPDH* Forward 5’‐AGCCTCAAGAT CATCAGC‐3’, Reverse 5’‐GAGTCCTTCCACGATACC‐3’. *GAPDH* served as the housekeeping gene. The relative gene expression was calculated using the 2^−ΔΔCt^ method. Each sample was run minimally at three concentrations in triplicate.

### 2.10 Tumorigenicity

A tumor xenograft model was conducted in six‐week‐old female BALB/c nude mice (18–22 g) that were obtained from the Chinese Academy of Sciences Shanghai Experimental Animal Center. BALB/c mice were maintained under controlled conditions with a 12 h light‐dark cycle, at 25–27℃, ∼40% humidity with access to food and water. Mice were subcutaneously inoculated into the flank with 1 × 10^6^ SKOV3 cells. All mice were randomly divided into four groups after one‐week inoculation; vehicle only group, Cyp (10 mg/kg) group, CQ (50 mg/kg) group, and Cyp (10 mg/kg)+CQ (50 mg/kg) group. Mice were injected by intraperitoneal (i.p.) every other day for 4 weeks. The tumor growth parameters were recorded. This study was approved by the institutional animal care and use committee of the Sir Run Run Shaw Hospital, School of Medicine, Zhejiang University.

### 2.11 Statistical analysis

Statistical analysis was performed using GraphPad Prism (version 5.0, GraphPad Software, Inc.). Quantitative data were recorded as means ± SD, while the IHC Scores were expressed as the median with range. Differences between groups were assayed using Student's *t* test or ANOVA tests for tumor growth, CCK‐8, Transwell assay; Differences between groups were assayed using Mann‐Whitney or Kruskal‐Wallis tests for the percentage of mCherry+GFP+, Wound healing assay and *qRT‐PCR*. The IHC scores variance was analyzed by Kruskal‐Wallis H test, two‐tailed. The correlation was calculated using the Pearson correlation coefficient. A *p*‐value of less than .05 was considered statistically significant. It only showed as <.0001 without a precise value when it was smaller than .0001.

## 3 RESULTS

### 3.1 Up‐regulation of SHH and accumulation of SQSTM1/P62 in epithelial ovarian cancer

**FIGURE 1:**
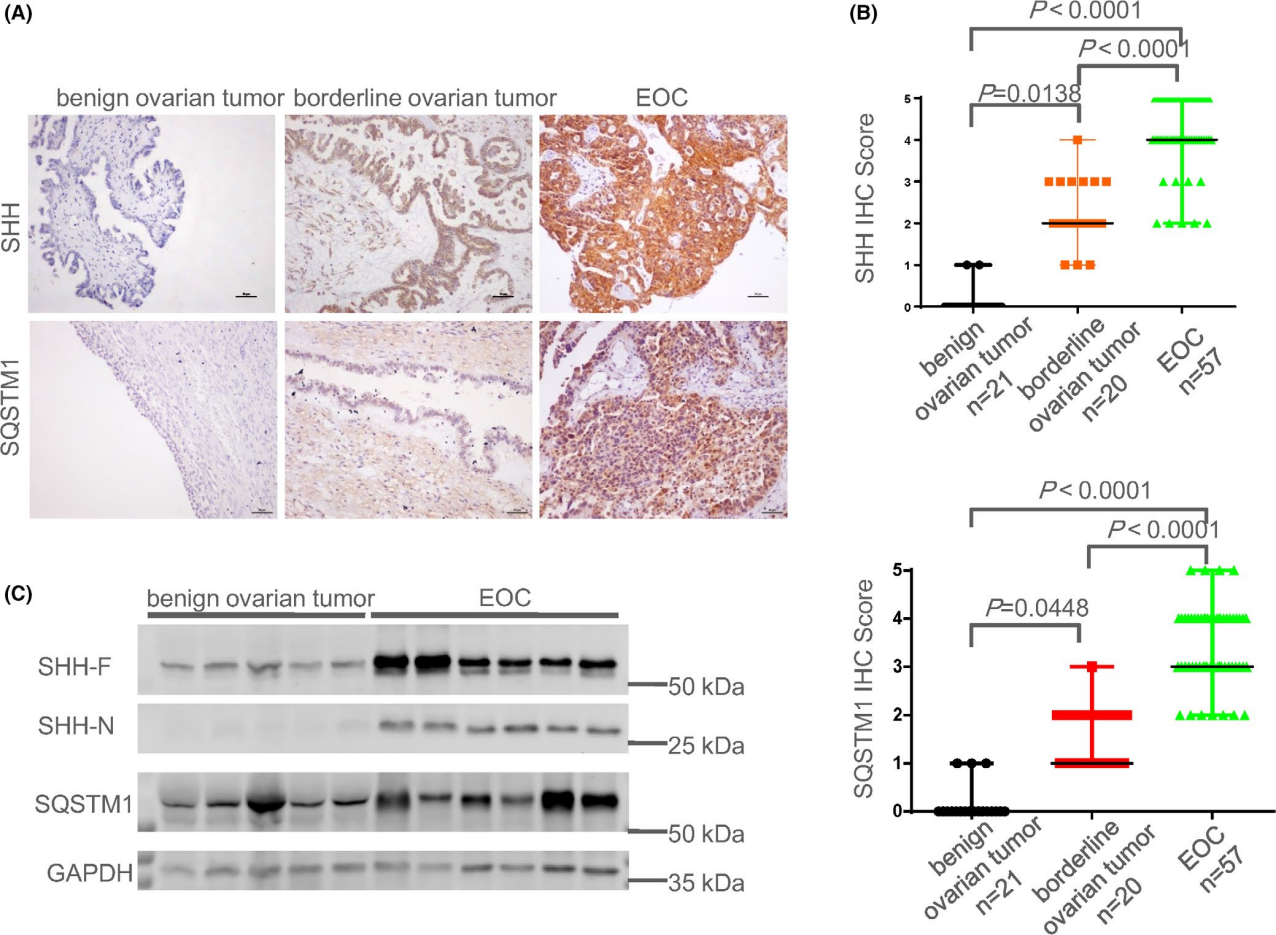
Up‐regulation of SHH and accumulation of SQSTM1/P62 in EOC. (A) The expressions of SHH and SQSTM1 in benign ovarian tumor, borderline ovarian tumor, and EOC were detected by IHC. Scale bars: 50 μm. (B) The IHC Scores of SHH and SQSTM1 in benign ovarian tumor (*n* = 21), borderline ovarian tumor (*n* = 20), and EOC (*n* = 57) by Kruskal‐Wallis H test, two‐tailed. Dots indicate the score of individuals. The line indicates the median with range. The intact data can be found in Table S1. (C) The protein expression levels of SHH and SQSTM1 were measured by Western Blot in malignant neoplasms and normal ovarian tissues

To determine whether the SHH signaling pathway is involved in the tumorigenesis and development of ovarian cancer, immunohistochemistry (IHC) was performed to identify the positive expressions of SHH and SQSTM1, a marker of autophagy inhibition^28^ in borderline ovarian tumor tissues, epithelial ovarian cancer (EOC) tissues and benign ovarian tumor tissues. The positive staining of SHH and SQSTM1 was reflected by brown granules in the cytoplasm. As shown in Figure 1A, SHH and SQSTM1 were found to be highly expressed in the samples of borderline ovarian tumors, and even higher in epithelial ovarian cancer (EOC), compared with those of benign ovarian tumors. Meanwhile, IHC scores of SHH and SQSTM1 were higher in borderline ovarian tumors and even higher than those in benign ovarian tumors (Figure 1B; Figure S3). In line with the results of the IHC, the protein expression levels of SHH‐F (precursor of the active SHH protein), SHH‐N, and SQSTM1 were dramatically increased in EOC tissues, compared to benign ovarian tumor tissues (Figure 1C). But, the sizes of SHH‐F and SHH‐N are higher than expected. We also found increased SHH‐F expression was associated with increased SQSTM1 with correlation coefficients of 0.3435, *p* = .0089 (Table S7). Collectively, these data suggest that the SHH pathway is activated and autophagy is disrupted in EOC.

### 3.2 Inhibition of SHH pathway induced autophagy in ovarian cancer cells

**FIGURE 2:**
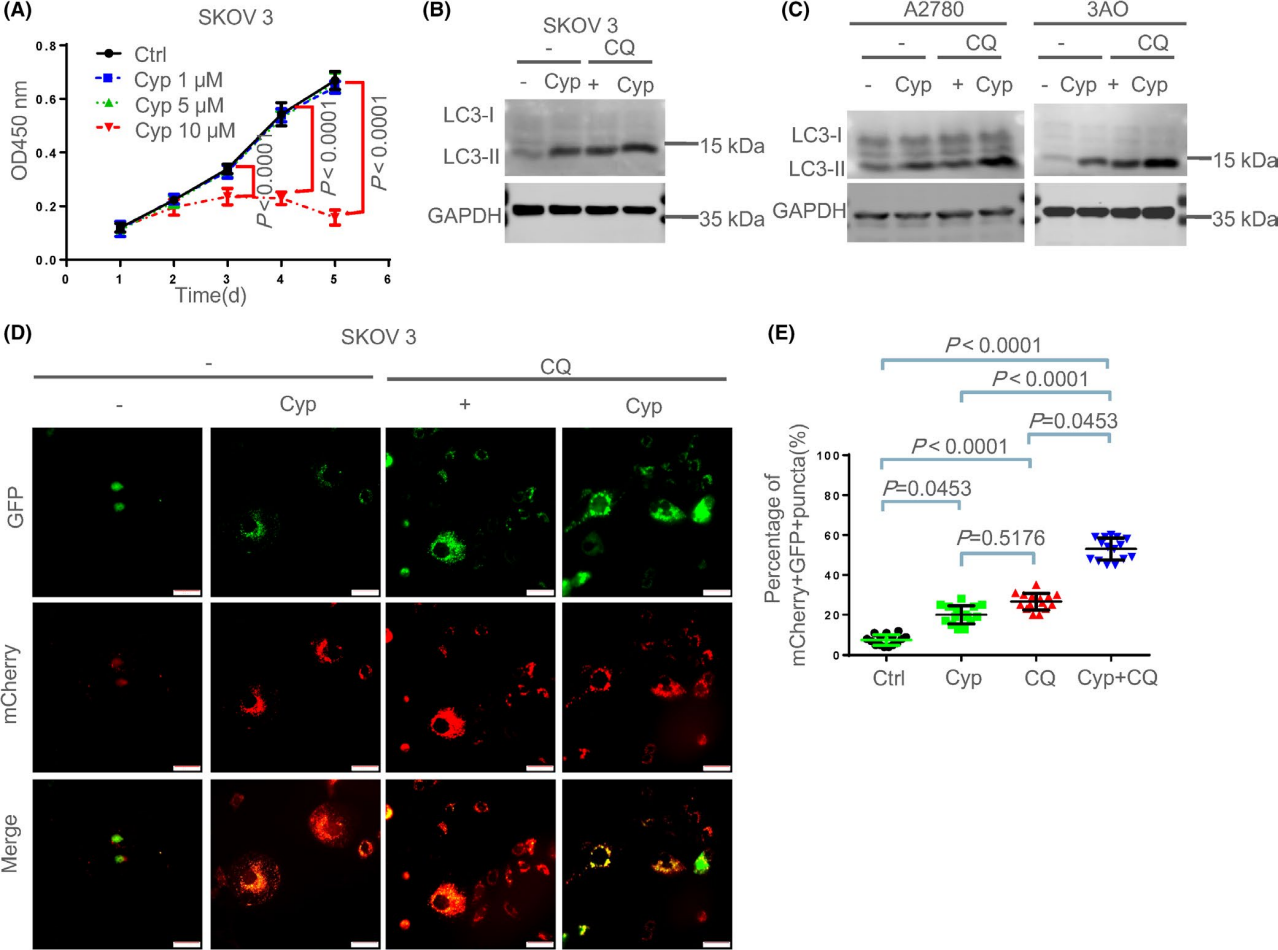
Inhibition of SHH pathway induced autophagy in ovarian cancer cells. (A) SKOV3 cells were treated with various concentrations of Cyp (1, 5 and 10 μM) for 1, 2, 3 (Cyp 10 μM versus Ctrl, *p* = 0.0012), 4 (Cyp 10 μM versus Ctrl, *p* < 0.0001) and 5 (Cyp 10 μM versus Ctrl, *p* < 0.0001) days and then cell viability was determined by CCK‐8 assay. These data are presented as means ± SD of three independent experiments, by two‐way ANOVA test with repeated measurement Tukey's post‐hoc test. The intact data can be found in Table S2. (B) SKOV3 cells were treated with 5 μM Cyp for 48 h with or without 5 μM chloroquine (CQ) (the pharmaceutical inhibitor of autophagy) for the last 4 h and then the LC3I/LC3II expression levels analyzed by WB. (C) A2780 and 3AO cells were treated with 5 μM Cyp for 48 h with or without 5 μM CQ for the last 4 h and then analyzed by WB respectively. (D, E) Analysis of mCherry‐GFP‐LC3 expressed in SKOV3 treated with 5 μM Cyp for 48 h with or without 5 μM CQ. mCherry‐positive GFP‐positive (mCherry+GFP+) puncta indicates autophagosomes, which were quantified by counting in five different fields from three independent experiments. Scale bars: 50 μm. The percentage of mCherry+GFP+ was quantified, followed by the Kruskal‐Wallis test. Data are shown as mean ± SD. The intact data can be found in Table S3. Each representative figure was obtained from three independent experiments

To interfere with the SHH pathway, we used Cyclopamine (Cyp), a well‐known inhibitor of SHH signaling pathway, which can block the activation of SHH pathway by binding directly to Smoothened (SMO).^29^ Effects of cyclopamine treatment after 48 h were tested by CCK‐8 assay as a sensitive measure of cell proliferation. The results showed that 5, 10 μM of Cyp could significantly reduce the relative expression of *Ptch1* and *GLI1* mRNA, indicating that it can block the activation of the SHH signaling pathway (Figure S1; Table S8). To exclude the possibility that Cyp treatment could affect the survival of SKOV3 cells, we screened the appropriate concentration and time by CCK8 assay. To this end, SKOV3 cells were treated with different concentrations of Cyp (1, 5, and 10 μM) for 1, 2, 3, 4, and 5 days. It was observed that the SKOV3 cells treated with 1 and 5 μM had no obvious tumor growth suppression from 1 to 5 days, while 10 μM Cyp treatment resulted in a significant reduction of the cell proliferation from 2 days, compared with the control group (Figure 2A; Table S2). So treatment with 5 μM of Cyp for 2 days was selected as the optimal condition for subsequent experiments.

Since the activity of the SHH pathway is notably related to autophagy activity, we sought to detect whether autophagy was upregulated in ovarian cancer after Cyp treatment. First, we determined the autophagy‐marker MAP1LC3B‐II^30^ in Cyp‐treated SKOV3 cells. We found that the expression of MAP1LC3B‐II protein was significantly increased in Cyp alone group compared to the control group. Additionally, the cultured SKOV3 cells were treated with 5 μM Cyp for 48 h and 5 μM chloroquine (CQ) (the pharmaceutical inhibitor of autophagy) for the last 4 h, and the expression of autophagic marker protein MAP1LC3B‐II were examined. It was shown that the expression levels of MAP1LC3B‐II protein were also significantly increased in CQ alone group compared to the control group. Interestingly, CQ combined with Cyp resulted in a further increase in the MAP1LC3B‐II protein levels (Figure 2B, Figure S5), implying an increased autophagic activity was probably due to the suppression of the SHH pathway. Cyp can unexpectedly increase the SQSTM1 protein and mRNA levels (Figure S2A,B; Table S8). The protein expression levels of MAP1LC3B‐II were also found to be significantly increased in A2780 and 3AO cells treated with Cyp and CQ compared with the control group (Figure 2C; Figure S4).

To further verify the role of the SHH pathway blockage in the induction of autophagy, autophagic flux was also observed. SKOV3 cells were transferred with Ad‐mCherry‐GFP‐LC3B adenovirus at a MOI of 80 for 48 h, followed by Cyp (5 μM) for 48 h with or without CQ (5 μM) for 4 h. When Ad‐mCherry‐GFP‐LC3 was used to track LC3 expression in autophagosomes and autolysosomes, we observed an increase of both yellow LC3 puncta (representing autophagosomes) and red puncta (representing autolysosomes) in the Cyp group, suggesting that Cyp could promote autophagosomes formation and enhance autophagy flux (Figure 2D,E; Table S3). Notably, CQ treatment led to a significant increase in the yellow dots, while it did not affect the number of red dots, implying autophagic degradation was blocked. It was also observed that CQ and Cyp co‐treatment resulted in a significant increase in the numbers of yellow dots, compared with Cyp alone or CQ alone group. Collectively, all data suggest that inhibition of the SHH signaling pathway by Cyp can activate autophagy in ovarian cancer cells.

### 3.3 Inhibition of the SHH pathway induced autophagy through the PI3K/AKT dependent pathway in ovarian cancer cells

**FIGURE 3:**
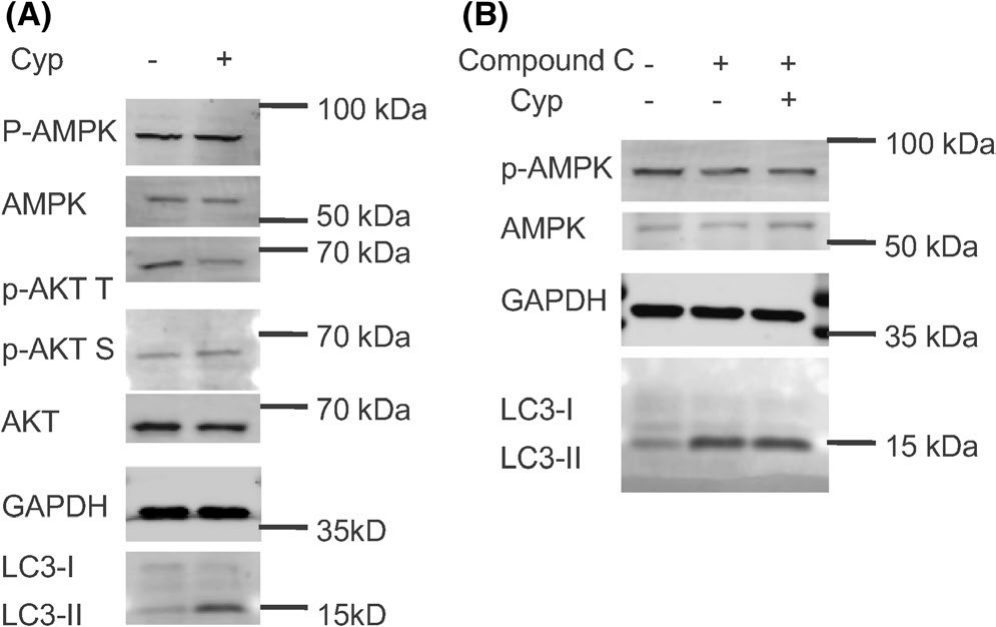
Cyp induced autophagy through a PI3K/AKT dependent pathway in ovarian cancer cells. (A) The cultured SKOV3 cells were treated with Cyp (5 μM) at 48 h. The expression levels of p‐AMPK, AMPK, p‐AKT T, and AKT were analyzed by Western Blot. (B) SKOV3 cells were treated with Cyp (5 μM) for 48 h, followed by compound C (16 μM) treatment, an inhibitor of AMPK for 12 h. The expression level of p‐AMPK was analyzed by Western Blot

It has been accepted that autophagy is regulated by various signaling pathways, in which AMPK and the PI3K/AKT signaling pathway have been widely studied.^31^, ^32^, ^33^, ^34^ Therefore, we sought to determine whether the observed inhibition of SHH pathway‐induced autophagy was mediated through AMPK/mTOR or the PI3K/AKT signaling pathways. Interestingly, the phosphorylation of p‐AKT T (Thr308) was significantly decreased, while the levels of p‐AKT S (Ser473), AKT, p‐AMPK, and AMPK have no obvious changes after Cyp treatment in comparison with the control group (Figure 3A; Figures S5 and S6).

To further confirm that Cyp‐induced autophagy is not dependent on the AMPK pathway, SKOV3 cells were treated with Cyp for 48 h, followed by compound C(16 μM) treatment, an inhibitor of AMPK for another 12 h. As expected, the phosphorylation of p‐AMPK was markedly decreased in the compound C alone group and compound C plus Cyp group, while the expression levels of AMPK have no obvious changes (Figure 3B; Figure S6). Meanwhile, we found that compound C treatment had no effect on the LC3II accumulation caused by Cyp induced in SKOV3 cells. All these results indicate that inhibition of the SHH pathway induced autophagy through the PI3K/AKT dependent pathway in ovarian cancer cells.

### 3.4 Inhibition of the SHH pathway promoted the migration of ovarian cancer cells through induction of autophagy

**FIGURE 4:**
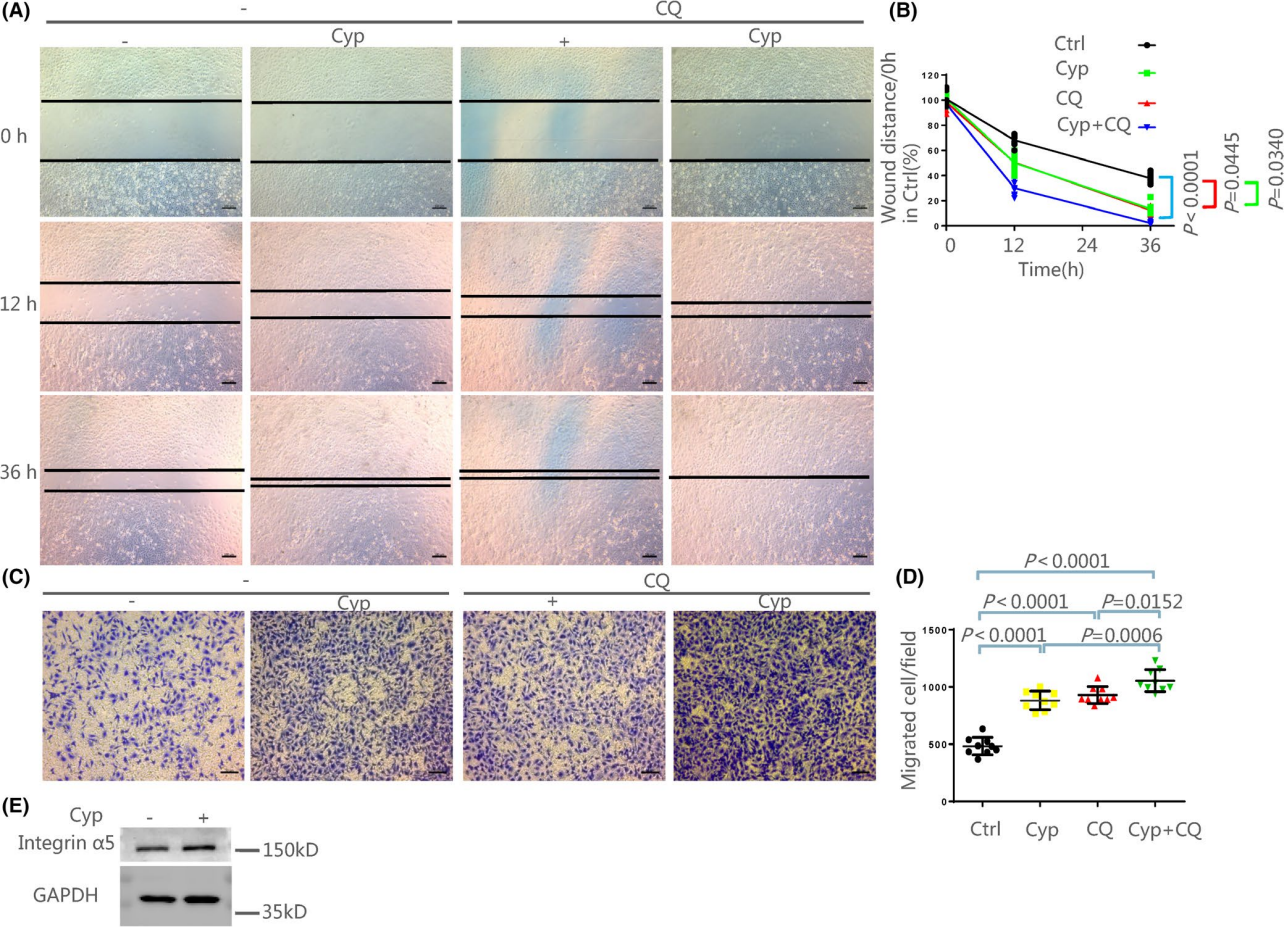
Cyp promoted the migration and invasion of ovarian cancer cells through induction of autophagy. The cultured SKOV3 cells were treated with 5 μM Cyp at 48 h after 5 μM CQ treatment and incubated for 4 h. (A, B) The migration of SKOV3 cells was detected by Wound‐healing assay. The wound distance of Ctrl at 0 h was defined as 100%. The percentage of wound distance was quantified, followed by the Kruskal‐Wallis test. Scale bars: 100 μm. Data are shown as mean ± SD, *n* = 3 independent experiments. The intact data can be found in Table S4. (C, D) The migration of SKOV3 cells was detected by the Transwell migration assay. The quantify of migrated cells was counted by Image J in three different visual fields, followed by the One‐way ANOVA test and Tukey's post‐hoc test. Scale bars: 50 μm. Data are shown as mean ± SD, *n* = 3 independent experiments. The intact data can be found in Table S5. (E) The expression of Integrin α5 was measured by Western Blot

To further investigate whether autophagy induced by inhibition of the SHH pathway could affect tumor migration, the cultured SKOV3 cells were treated with 5 μM Cyp followed by 5 μM CQ treatment. The wound‐healing assay showed that the wound healing area in Cyp or CQ alone group was smaller than that in the control group. Notably, CQ treatment enhanced the promoting effect of Cyp on the wound‐healing area (Figure 4A,B; Table S4). Transwell assay showed that Cyp or CQ alone treatment promoted the migratory ability of SKOV3 cells, compared to the control group, and the promoting effect of Cyp on the cell migration was enhanced when SKOV3 cells were co‐treated with 5 μM Cyp and 5 μM CQ (Figure 4C,D; Table S5). Correspondingly, it was observed that the expression of Integrin α5, which is known to regulate migration,^35^ was significantly increased in Cyp alone group, compared to the control group (Figure 4E; Figure S7). All data suggest that autophagy contributes to ovarian cancer migration induced by inhibition of the SHH pathway.

### 3.5 The combination of Cyp and CQ suppressed ovarian cancer cells growth in vivo

**FIGURE 5:**
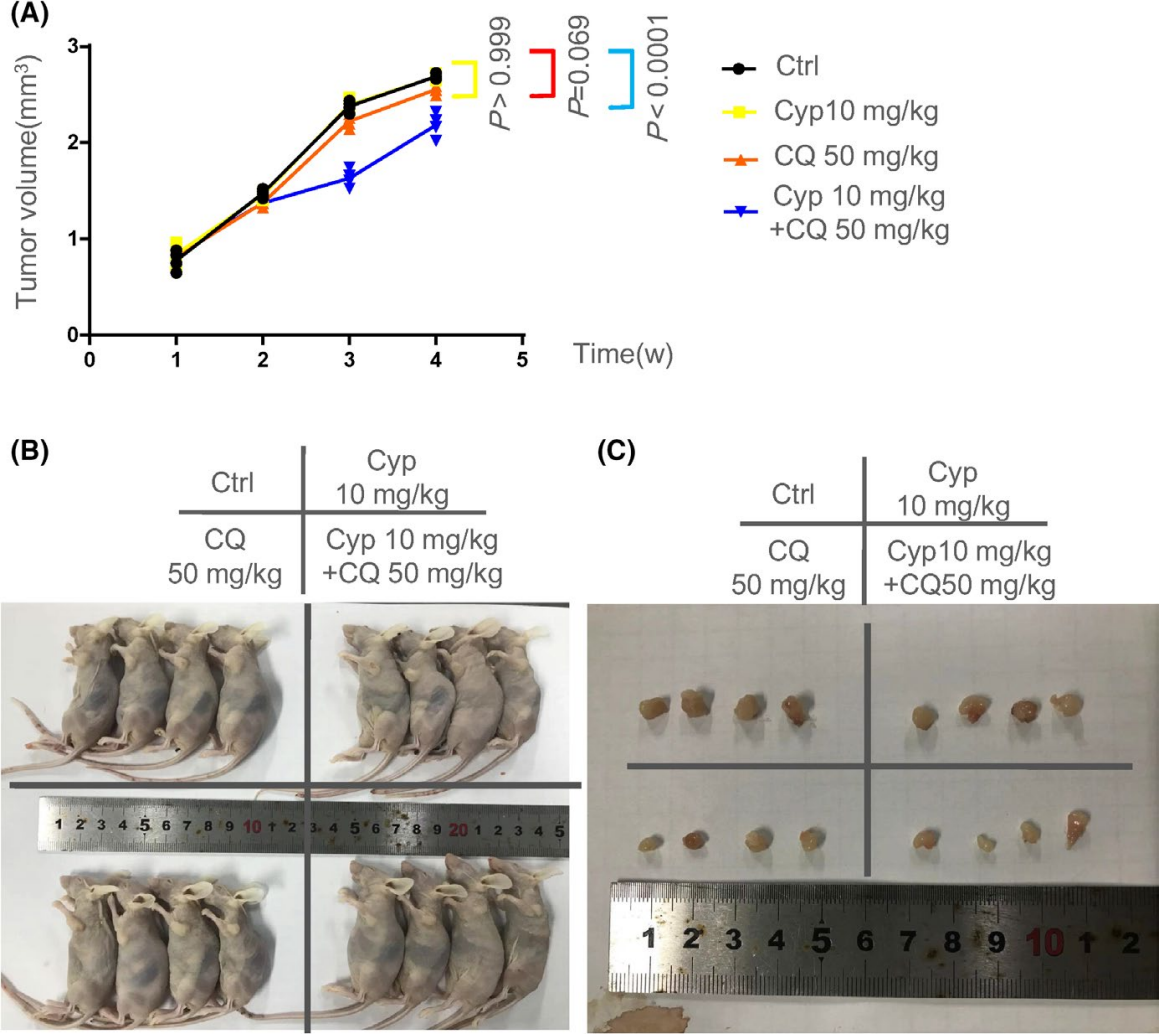
Tumor suppressive role of Cyp and CQ in ovarian cancer in vivo. Mice were subcutaneously inoculated into a flank with SKOV3 cells, followed by treatment with Cyp or/and CQ by intraperitoneal (i.p.) injection every other day for 4 weeks. (A) Tumor volume at indicated time points in nude mice; 1 × 10^6^ SKOV3 cells/mouse; *n* = 4 mice per group. The tumor volume is shown as mean ± SD, followed by Two‐way ANOVA with repeated measurement and Tukey's post‐hoc test. The intact data can be found in Table S6. (B, C) Photography of xenograft tumor in nude mice

To further investigate the role of the SHH pathway and autophagy in vivo, SKOV3 cells were subcutaneously implanted into the flank of mice to establish xenografts, into which Cyp (10 mg/kg), CQ (50 mg/kg), or Cyp (10 mg/kg) in combination with CQ (50 mg/kg) were injected every other day for 4 weeks. The doses of CQ and Cyp were mainly determined by previous studies.^36^, ^37^ Our results showed that treating mice with both SHH inhibitor Cyp and autophagy suppressor CQ markedly suppressed the tumor growth, compared with Cyp or CQ alone group (Figure 5A; Table S6). After 28‐day observation, mice with xenografts were killed, and the tumors were then weighted (Figure 5B,C). Taken together, our results suggested that simultaneous inhibition of the SHH signaling pathway and autophagy inhibited tumor growth in vivo.

## 4 DISCUSSION

In the present study, we found that inhibition of the SHH pathway induced autophagy in ovarian cancer cells. Furthermore, inhibition of the SHH pathway promoted migration of ovarian cancer cells in vitro through induction of autophagy. Additionally, Cyp in combination with CQ inhibited tumor growth in vivo. Our findings suggest that targeting the SHH pathway and autophagy may be a potential therapeutic strategy for ovarian cancer patients.

Increasing evidence has reported the interaction between the SHH signaling pathway and autophagy in various types of cancer cells. For instance, Milla et al. showed that SHH antagonist Cyp subdued autophagy levels in SHSY5Y cells, a neuroblastoma cell line.^38^ Lo Ré et al. showed that autophagy is induced by GLI3, a well‐known nuclear executor of the SHH pathway, which regulated the expression and promoter activity of VMP1(Lo,^39^ Wang et al. found that in HCC cells, suppression of the SHH pathway promoted autophagy through up‐regulating the expression of Bnip3.^25^ In the contrast, a recent study demonstrated that SHH signaling prevented the induction of autophagy in an ovarian cancer cell line, ES2.^40^ These previous findings suggest that a paradoxical relationship exists between the SHH signaling pathway and autophagy. In our study, the significant upregulation of SHH and SQSTM1 was observed in borderline and EOC, suggesting the activation of SHH signaling and inhibition of autophagy in ovarian cancer. Notably, SHH and SQSTM1 staining are not in the same cells‐‐SHH being predominantly in the epithelial part and SQSTM1 in the stroma, as evidenced by observation in the borderline samples, which points to paracrine signaling in the ovary. Using Cyp, a chemical compound that has been shown to inhibit SHH function by binding to SMO,^29^ we found that Cyp treatment resulted in a significant decrease in cell viability of ovarian cells at a high concentration (10 μM), whereas Cyp has no effect on the cell viability at a low concentration (5 or 1 μM). It is well‐known that when the cells are treated with lysosomotropic agents such as CQ, the degradation of MAP1LC3B‐II is blocked, which in turn resulted in the accumulation of MAP1LC3B‐II.^40^ Consistent with this, we found that the expression levels of MAP1LC3B‐II were significantly increased in 5 μM Cyp or 5 μM CQ alone treated ovarian cells, and Cyp combined with CQ synergistically enhanced the expression levels of MAP1LC3B‐II, consolidating the functional roles of the SHH signaling pathway in induction of autophagy. Interestingly, a previous study revealed that downregulation of the SHH pathway with GANT‐61 has proved to be more effective than Cyp in the context of ovarian cancer.^41^ Therefore, we will examine whether GANT‐61 has similar effects with Cyp on autophagy in the future.

Many studies have shown that many signal transduction pathways are involved in the regulation of autophagy, in which the AMPK signaling pathway has a key role.^42^, ^43^ Recent studies have demonstrated that AMPK is a regulator of autophagy and can activate tuberous sclerosis complex 1/2, leading to inhibition of the mTOR pathway and initiation of autophagy.^44^, ^45^, ^46^ In our study, we found that no matter Cyp, CQ, or Cyp plus CQ did not influence the expression of p‐AMPK. Meanwhile, the treatment of AMPK inhibitor (compound C) did not reverse Cyp mediated the induction of autophagy. The PI3K/AKT pathway is another important signal pathway involved in the regulation of autophagy.^47^ Previous studies have reported that inhibition of the AKT pathway and its downstream target mTOR contributes to the initiation of autophagy.^44^ Notably, SHH was also reported to stimulate the AKT pathway in several other cells.^48^, ^49^ Therefore, we propose that Cyp may induce the autophagy through PI3K/AKT pathway. To this end, the expressions of pathway‐related proteins and autophagy‐related signals were detected. The results demonstrated that low dose Cyp could suppress the expression levels of phosphorylated‐AKT in SKOV3 cells. These data suggest that low‐dose Cyp induces autophagy through inhibition of phosphorylated‐AKT in SKOV3 cells, but not the AMPK pathway.

Autophagy has been demonstrated to act as a key factor for the SHH signaling pathway functions, with marked effects on proliferation, apoptosis, and invasion of various types of cells, especially tumor cells.^50^, ^51^, ^52^ For example, Li et al. showed that SHH signaling promoted cell proliferation of vascular smooth muscle cells (VSMCs) depended on the activation of autophagy.^49^ Xu et al. found that SHH inhibition suppressed viability and induced apoptosis in pancreatic cancer both in vivo and in vitro through regulation of the autophagy.^53^ Therefore, we hypothesized that inhibition of the SHH signaling pathway may affect the progression of ovarian cancer through the regulation of autophagy, which has not been investigated previously. In this study, low dose Cyp could increase the migration ability of SKOV3 cells, and blockage of autophagy by CQ enhanced Cyp induced the migration ability in vitro. It was also observed that Cyp combined with CQ synergistically inhibited tumor growth in vivo. All these data suggest that inhibition of the SHH pathway may be an effective treatment strategy in ovarian cancer.

In other studies, Tong et al. have found autophagy can regulate epithelial‐mesenchymal transition (EMT) through TGF‐β1/Smad3 in bladder cancer cells^54^; Zheng et al. have found autophagy is an important mediator of migration in SKOV3 cells^55^; Zhan et al. have found autophagy can facilitate the migration of lung cancer cells.^56^ Many studies have proved that SQSTM1 can promote proliferation, invasion, and mesenchymal transition in many cells.^57^, ^58^, ^59^, ^60^, ^61^ Therefore, we supposed that Cyp and CQ can induce SQSTM1 to promote the migration and invasion in SKOV3 cells.

In conclusion, our findings reveal novel biological insight into how the SHH pathway regulates the migration of ovarian cancer cells and provides strong evidence that combined treatment with the SHH pathway inhibitor and autophagy inhibitor might be an effective therapeutic option in ovarian cancer therapy.

## CONFLICT OF INTEREST

The authors declare no competing financial interests.

## AUTHOR CONTRIBUTIONS

Yibin Pan performed data analysis and wrote the first draft of the article. Jiena Zhou contributed to the clinical specimen and data collections. Weidan Zhang, Meifei Lu and Yongdong Dai performed data analysis and interpretation. Lili Yan and Hanjing Zhou contributed to the WB experiment of Supplementary information when revising this manuscript. Songying Zhang, Jianhua Yang designed and directed the study.

## Supporting information

Fig S1

Fig S2

Fig S3

Fig S4

Fig S5

Fig S6

Fig S7

Fig S8

Table S1

Table S2

Table S3

Table S4

Table S5

Table S6

Table S7

Table S8
