# Supplementary figures and images for: The Sonic Hedgehog signaling pathway regulates autophagy and migration in ovarian cancer
Source: Cancer Med. 2021 Jun 2;10(13):4510–21. doi: 10.1002/cam4.4018 (PMC8267163; doi:10.1002/cam4.4018)

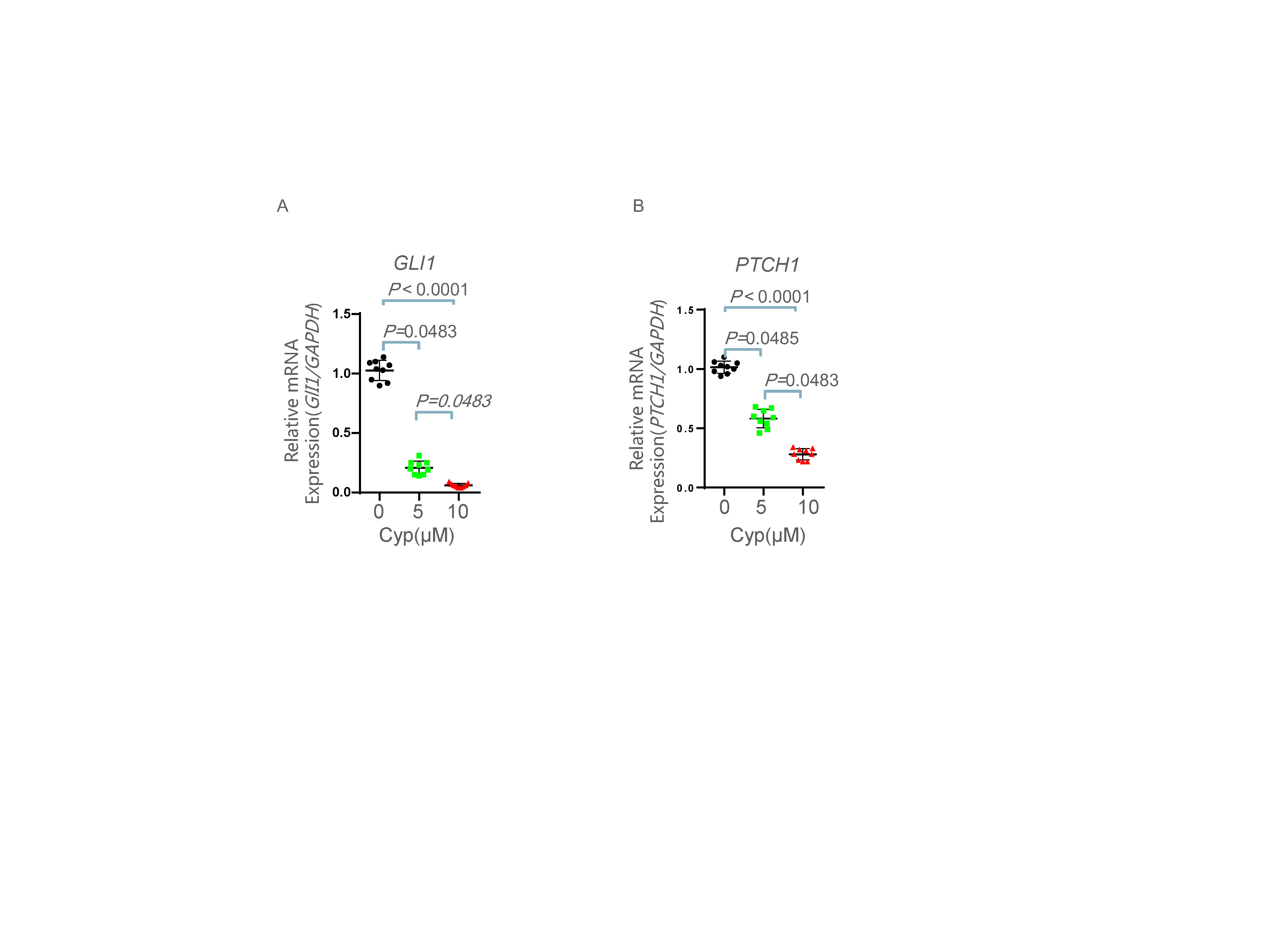

Supplement: Supplementary file 1 — Fig S1 [file CAM4-10-4510-s009.tiff]

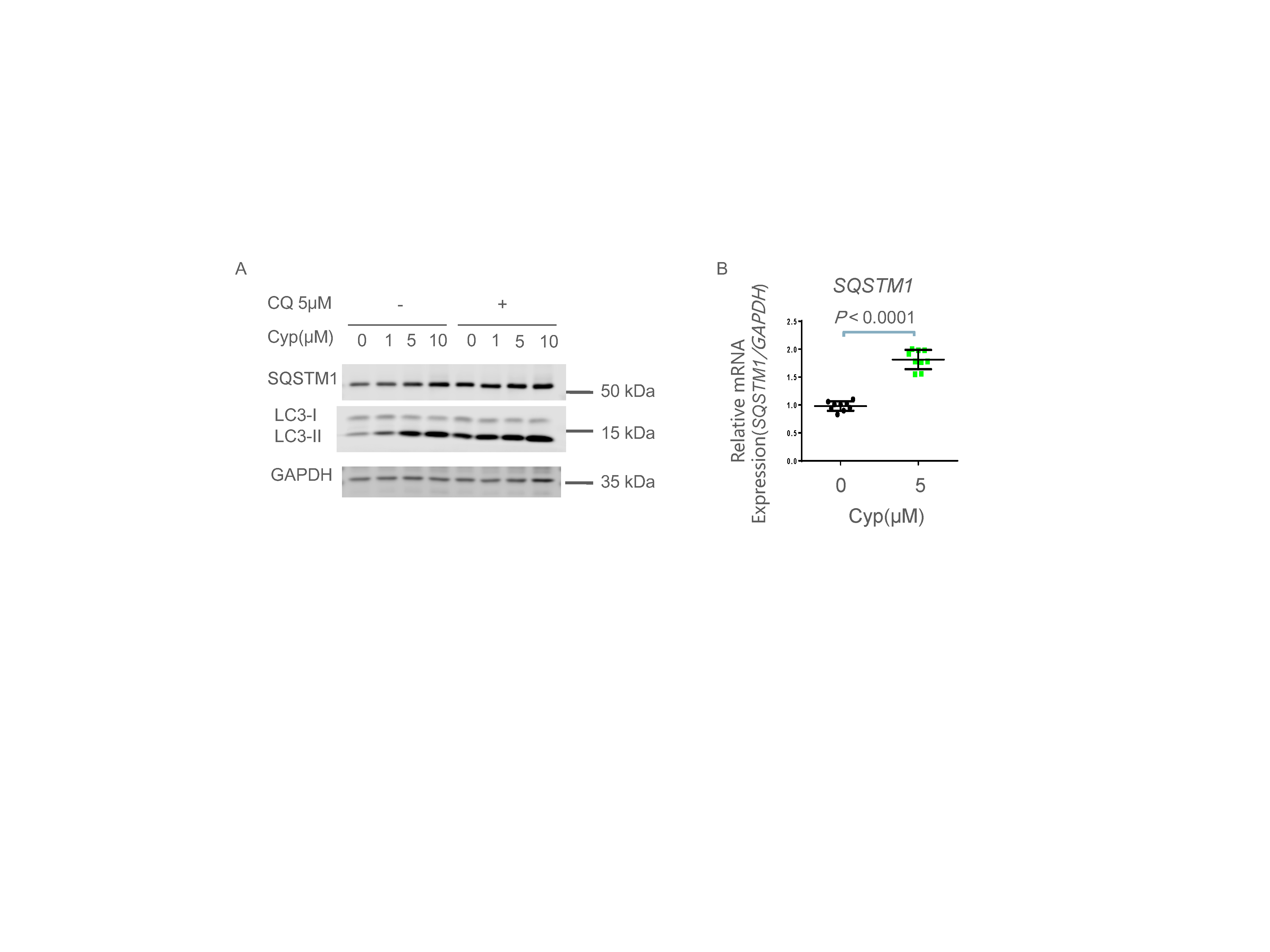

Supplement: Supplementary file 2 — Fig S2 [file CAM4-10-4510-s003.tiff]

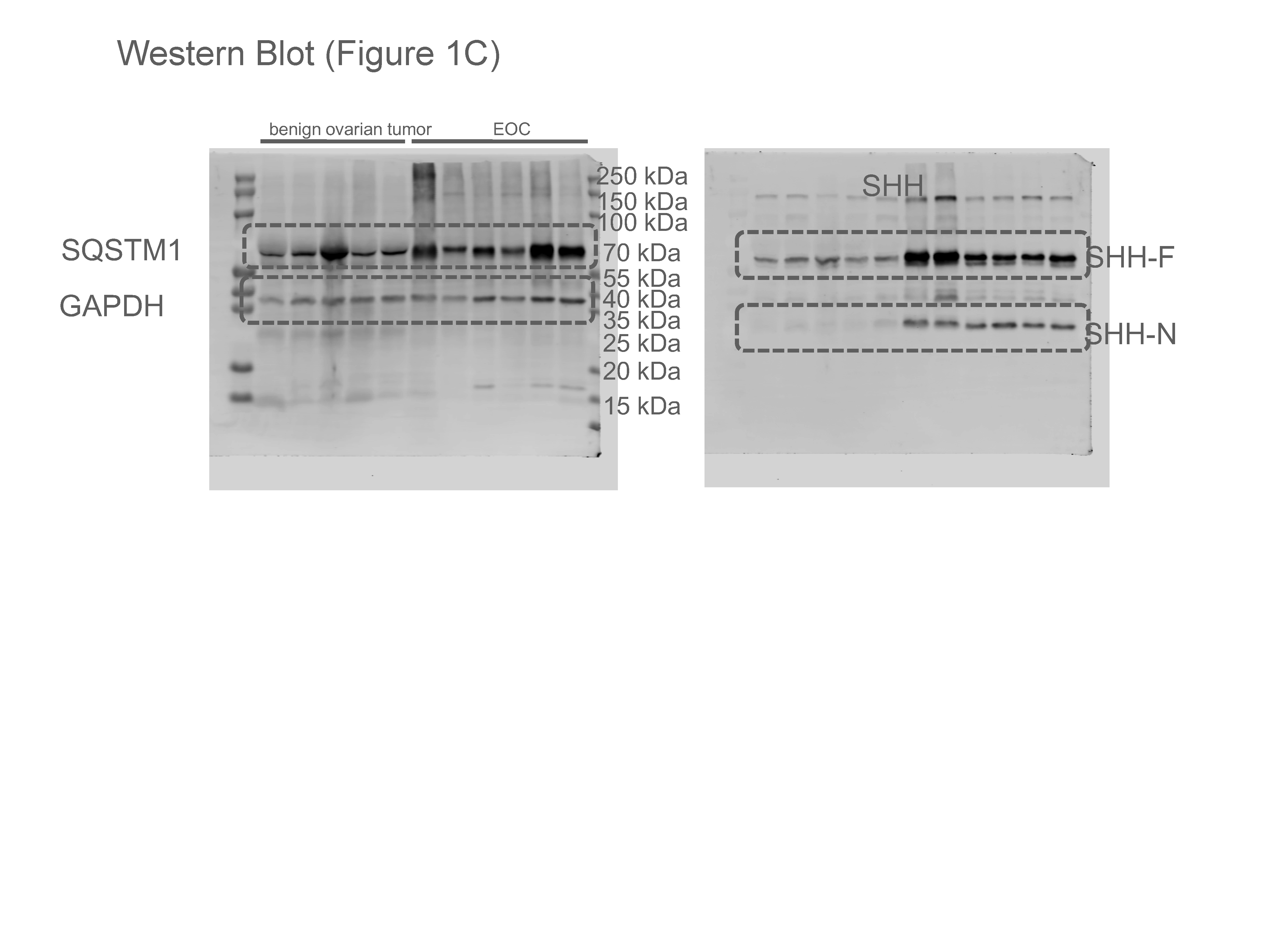

Supplement: Supplementary file 3 — Fig S3 [file CAM4-10-4510-s006.tiff]

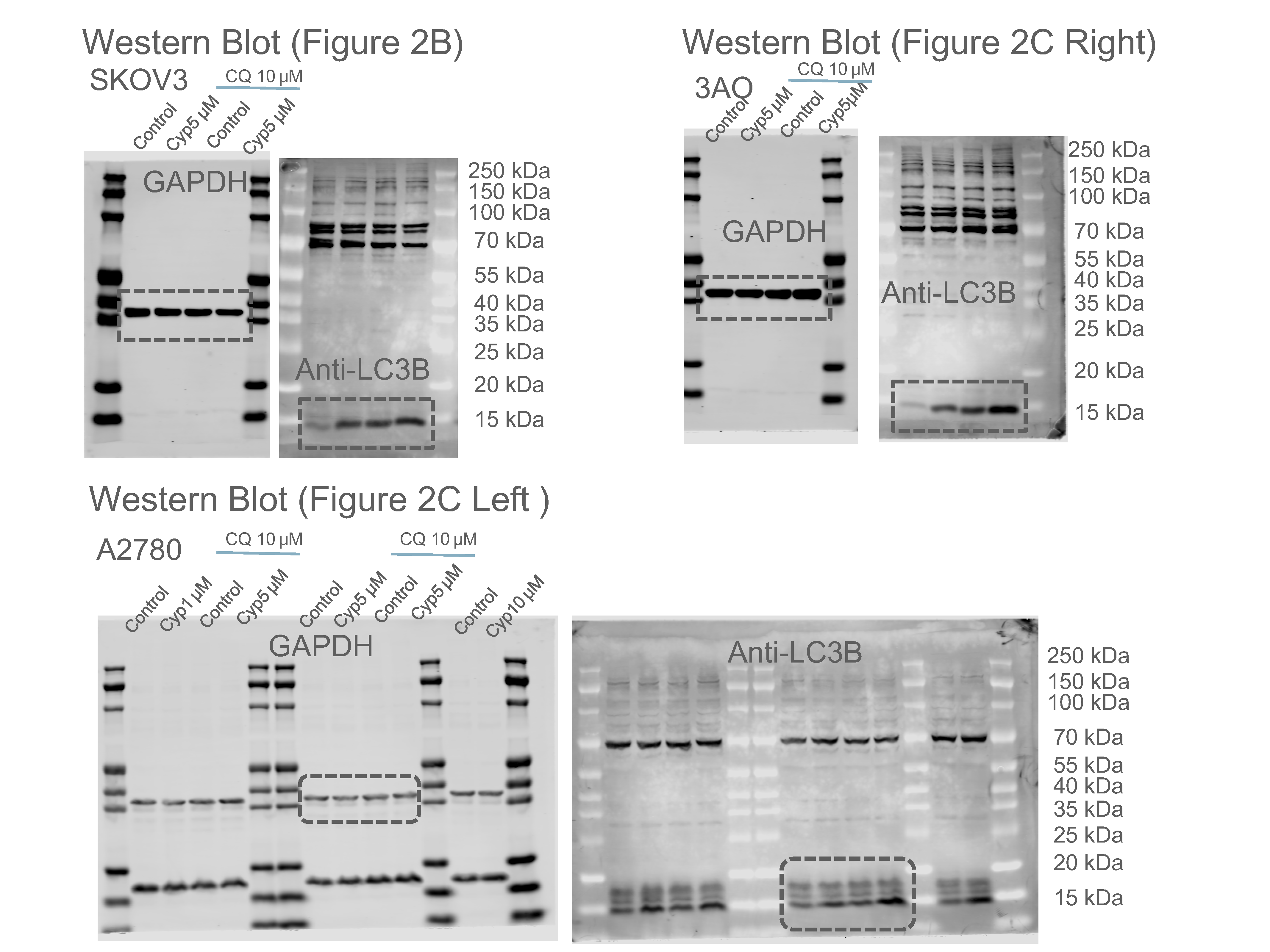

Supplement: Supplementary file 4 — Fig S4 [file CAM4-10-4510-s004.tiff]

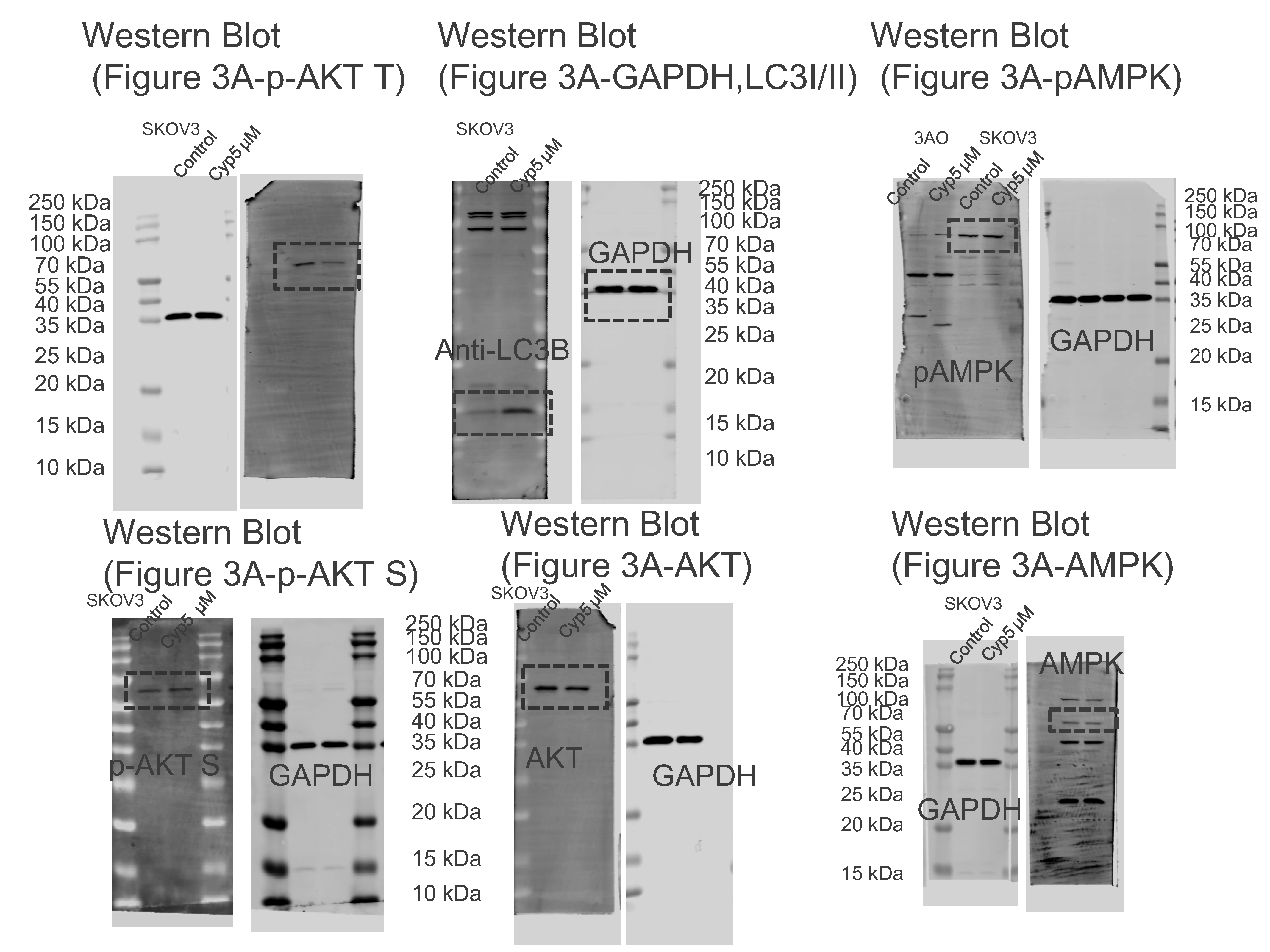

Supplement: Supplementary file 5 — Fig S5 [file CAM4-10-4510-s016.tiff]

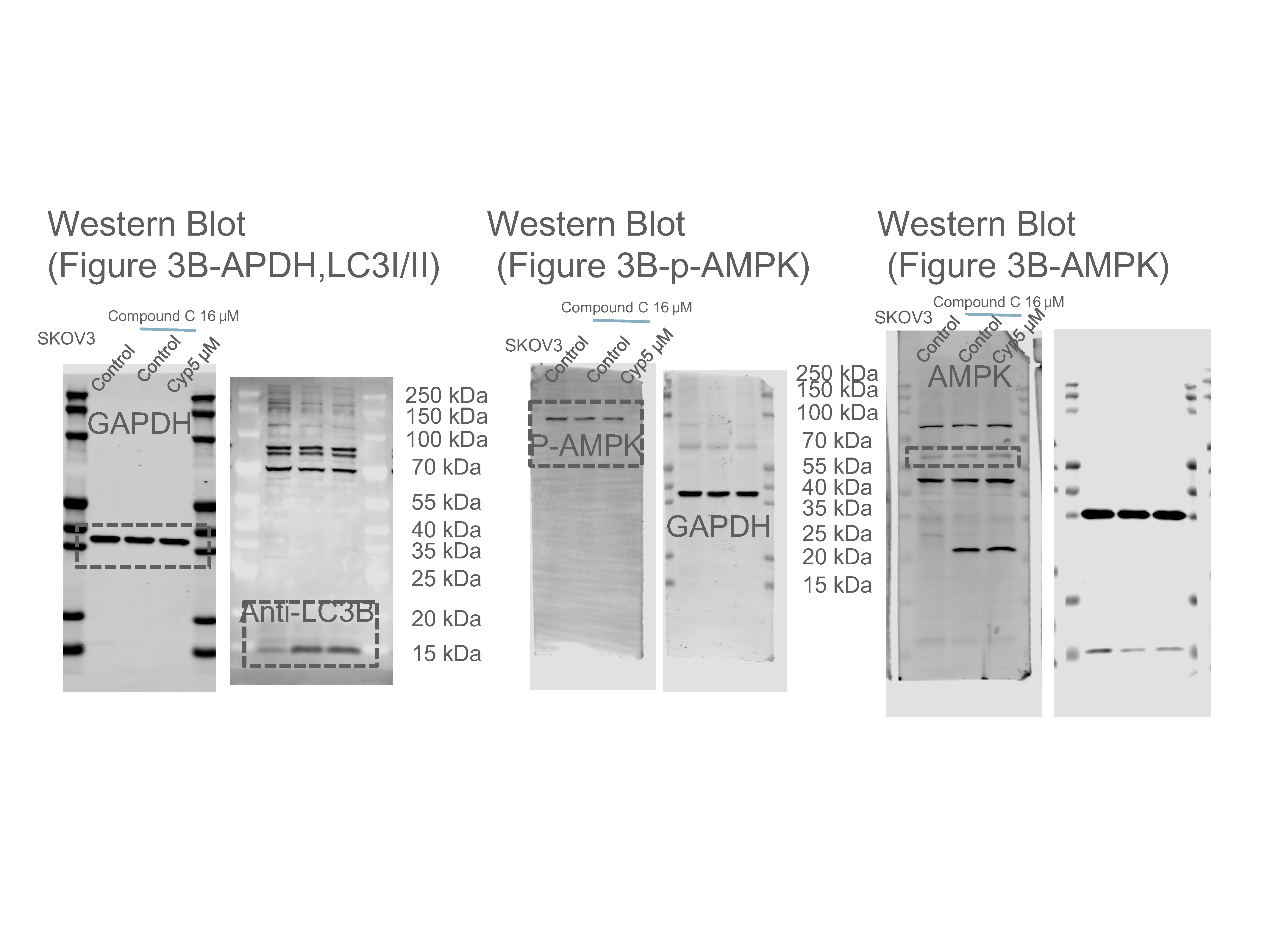

Supplement: Supplementary file 6 — Fig S6 [file CAM4-10-4510-s013.tiff]

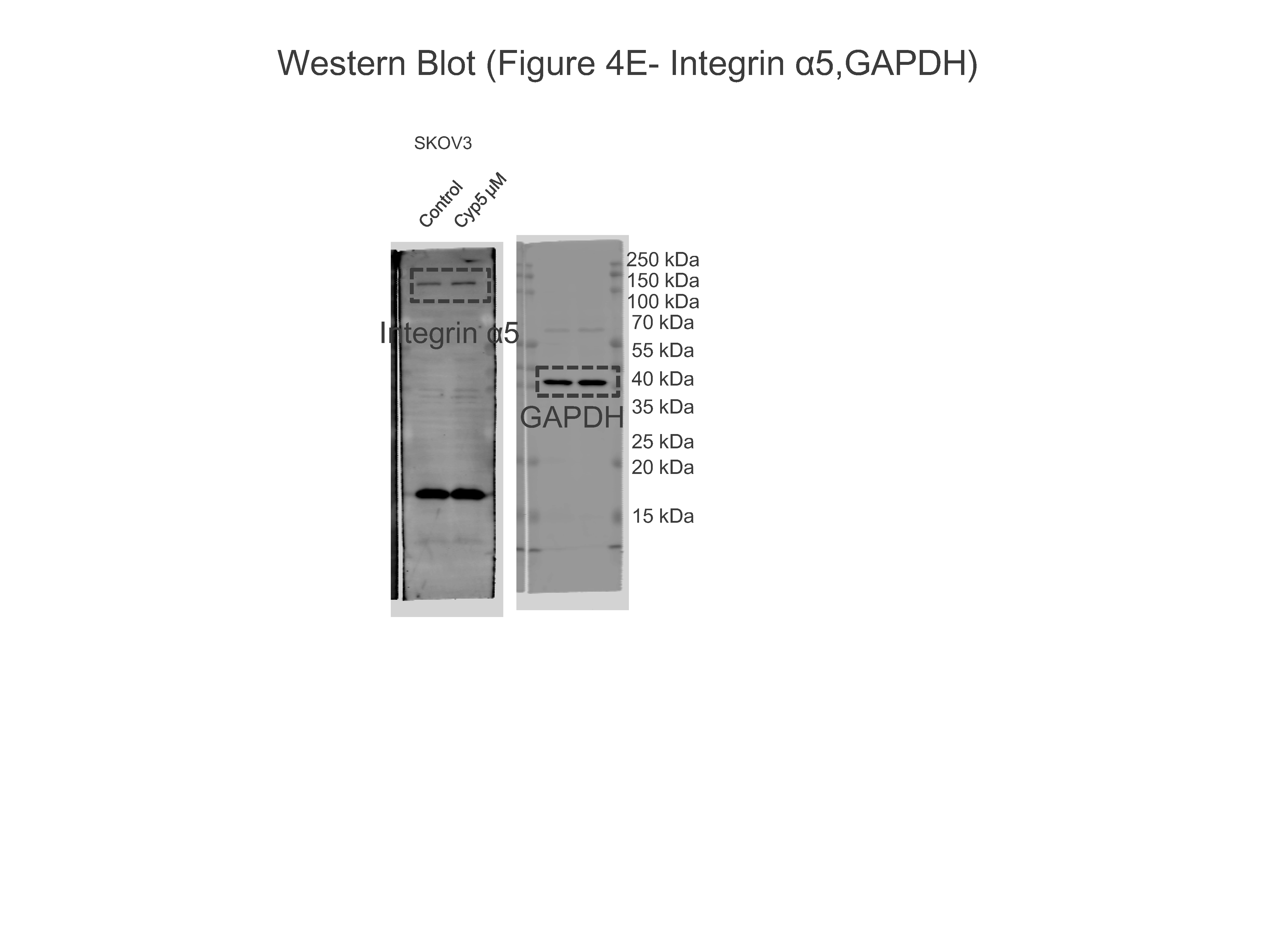

Supplement: Supplementary file 7 — Fig S7 [file CAM4-10-4510-s011.tiff]

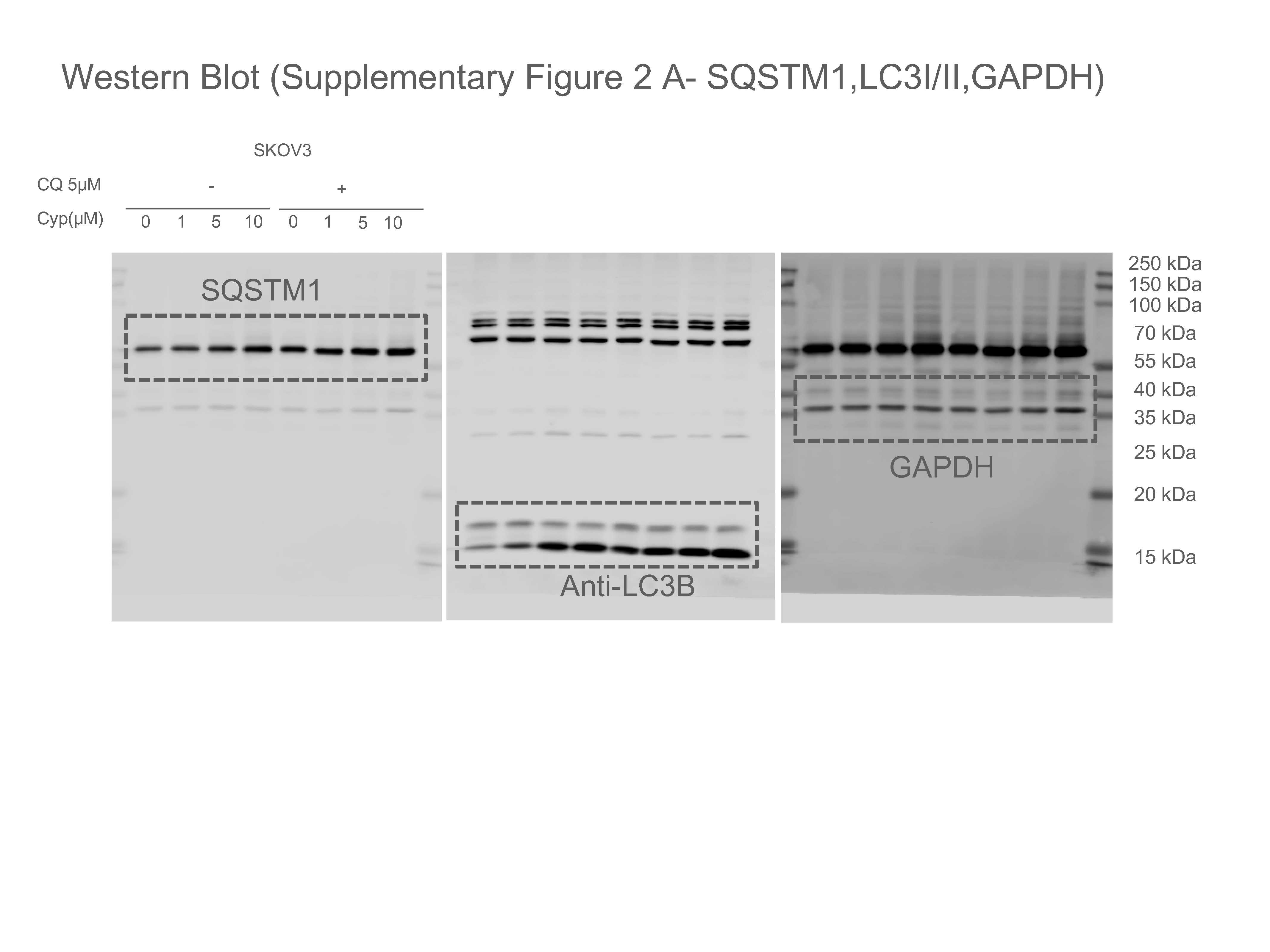

Supplement: Supplementary file 8 — Fig S8 [file CAM4-10-4510-s014.tiff]
